# Effect of Melatonin on the Renin-Angiotensin-Aldosterone System in l-NAME-Induced Hypertension

**DOI:** 10.3390/molecules23020265

**Published:** 2018-01-29

**Authors:** Fedor Simko, Tomas Baka, Kristina Krajcirovicova, Kristina Repova, Silvia Aziriova, Stefan Zorad, Marko Poglitsch, Michaela Adamcova, Russel J. Reiter, Ludovit Paulis

**Affiliations:** 1 Institute of Pathophysiology, Faculty of Medicine, Comenius University, Sasinkova 4, 81108 Bratislava, Slovakia; tomasko.baka@gmail.com (T.B.); krikratina@gmail.com (K.K.); repova.k@gmail.com (K.R.); silvia.aziriova@gmail.com (S.A.); ludovit.paulis@gmail.com (L.P.); 2 3rd Department of Internal Medicine, Faculty of Medicine, Comenius University, 83305 Bratislava, Slovakia; 3 Institute of Experimental Endocrinology, Biomedical Research Center, Slovak Academy of Sciences, 84505 Bratislava, Slovakia; Stefan.Zorad@savba.sk; 4 Attoquant Diagnostics, 1030 Vienna, Austria; marko.poglitsch@attoquant.com; 5 Department of Physiology, School of Medicine, Charles University, 50003 Hradec Kralove, Czech Republic; adamcova@lfhk.cuni.cz; 6 Department of Cellular and Structural Biology, UT Health Science Center, San Antonio, TX 78229, USA; reiter@uthscsa.edu; 7 Institute of Normal and Pathological Physiology, Center for Experimental Medicine, Slovak Academy of Sciences, 81371 Bratislava, Slovakia

**Keywords:** l-NAME, fibrosis, melatonin, angiotensin II, angiotensin 1–7, aldosterone

## Abstract

The renin-angiotensin-aldosterone system (RAAS) is a dominant player in several cardiovascular pathologies. This study investigated whether alterations induced by l-NAME, (NLG)-nitro-l-arginine methyl ester, a nitric oxide synthase inhibitor, and the protective effect of melatonin are associated with changes in the RAAS. Four groups of 3-month-old male Wistar rats (*n* = 10) were treated as follows for four weeks: untreated controls, rats treated with melatonin (10 mg/kg/day), rats treated with l-NAME (40 mg/kg/day), and rats treated with l-NAME + melatonin. l-NAME administration led to hypertension and left ventricular (LV) fibrosis in terms of enhancement of soluble, insoluble and total collagen concentration and content. Melatonin reduced systolic blood pressure enhancement and lowered the concentration and content of insoluble and total collagen in the LV. The serum concentration of angiotensin (Ang) 1–8 (Ang II) and its downstream metabolites were reduced in the l-NAME group and remained unaltered by melatonin. The serum aldosterone level and its ratio to Ang II (AA2-ratio) were increased in the l-NAME group without being modified by melatonin. We conclude that l-NAME-hypertension is associated with reduced level of Ang II and its downstream metabolites and increased aldosterone concentration and AA2-ratio. Melatonin exerts its protective effect in l-NAME-induced hypertension without affecting RAAS.

## 1. Introduction

Nitric oxide (NO) provides cardiovascular protection by reducing blood pressure and via its antiproliferative and antifibrotic actions [1,2]. Chronic l-NAME ((NLG)-nitro-l-arginine methyl ester) administration causes hypertension and target organ damage via the inhibition of NO synthase activity reducing NO production [3,4,5]. Moreover, l-NAME-induced deterioration of the vasodilatory function of the renal artery may trigger renin release and renin-angiotensin-aldosterone system (RAAS) activation [6,7].

Melatonin (*N*-acetyl-5-methoxytryptamine), the secretory product of the vertebrate pineal gland, influences biological rhythms [8,9], it also has a number of beneficial effects on the cardiovascular system including blood pressure reduction and attenuation of target organ damage [10,11,12]. Conversely, melatonin deficiency induced by experimental pinealectomy or continuous light exposure in rats results in the development of hypertension and myocardial fibrosis [13,14]. The pathomechanism of melatonin protection seems to be highly complex. Besides the activation of specific membrane and nuclear receptors [15,16], melatonin provides cardiovascular protection on several levels including antioxidant and scavenging actions, endothelial protection or sympatholytic effects [13,17,18,19,20], and additional pleiotropic effects are emerging [21]. However, there is a shortage of data on the ability of melatonin to interfere with neurohumoral activation, particularly with the RAAS in variable cardiovascular pathologies. The aim of the current study was to test whether hemodynamic and structural changes induced by l-NAME are associated with the modification in the RAAS and whether melatonin interferes with RAAS alterations.

## 2. Results

### 2.1. Cardiovascular Parameters

**Figure 1:**
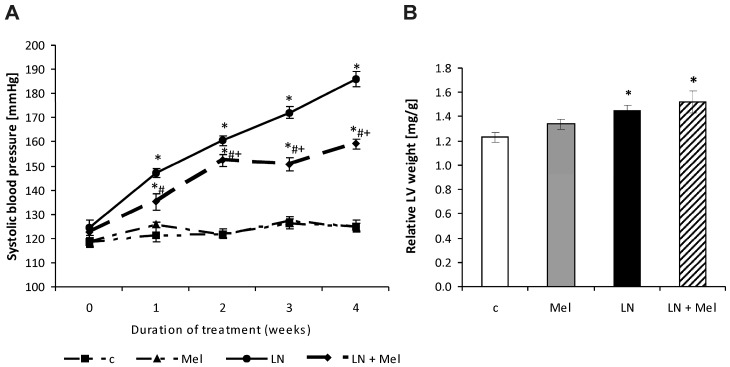
The influence of melatonin (LN + Mel) on blood pressure (**A**) and relative left ventricular weight (LVW/BW); (**B**) in l-NAME-treated rats. Wistar controls (c); l-NAME (LN); melatonin (Mel); *n* = 10 per group; * *p* < 0.05 vs. c; # *p* < 0.05 vs. LN; + *p* < 0.05 vs. Mel.

After four weeks of treatment, systolic blood pressure (SBP) was 124.68 ± 1.52 mmHg in the control and 185 ± 3.05 mmHg in l-NAME groups. SBP was significantly lower (*p* < 0.05) due to melatonin treatment (15%) compared to the l-NAME group (Figure 1A). The left ventricular weight/body weight (LVW/BW) ratio after four weeks of treatment was 1.229 ± 0.040 mg/g and 1.450 ± 0.043 mg/g, in the control and l-NAME-groups, respectively; this was not influenced by melatonin treatment (Figure 1B). The body weight (BW), absolute left ventricular weight (LVW) and right ventricular weight (RVW) and RVW/BW ratio are described in Table 1.

### 2.2. Hydroxyproline Concentration and Content in the Soluble, Insoluble and Total Collagenous Proteins

**Figure 2:**
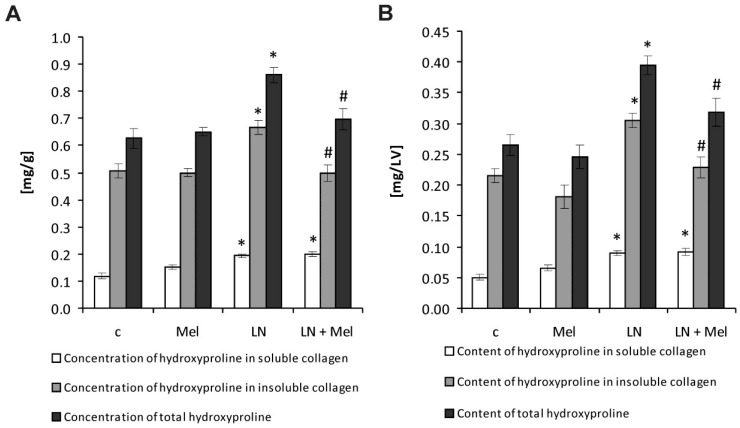
The influence of melatonin (LN + Mel) on hydroxyproline concentration in soluble and insoluble collagen proteins and on total hydroxyproline concentration (**A**) and content (**B**) in l-NAME-treated rats. Wistar controls (c); l-NAME (LN); melatonin (Mel); *n* = 10 per group; * *p* < 0.05 vs. c; # *p* < 0.05 vs. LN.

After four weeks of treatment, the hydroxyproline concentration in the soluble collagenous proteins in the control and l-NAME groups was 0.119 ± 0.011 mg/g and 0.194 ± 0.007 mg/g, respectively. Melatonin had no effect on hydroxyproline concentration in the soluble collagenous fraction (Figure 2A). The hydroxyproline concentration in the insoluble collagenous fraction in the control and l-NAME groups was 0.507 ± 0.027 mg/g and 0.665 ± 0.026 mg/g, respectively; this was significantly reduced by melatonin (25%, *p* < 0.05) (Figure 2A). The total hydroxyproline concentration in the control and l-NAME groups was 0.626 ± 0.038 mg/g and 0.860 ± 0.030 mg/g, respectively, and was reduced by melatonin (19%, *p* < 0.05) (Figure 2A). The hydroxyproline content in the soluble collagenous proteins in the control and l-NAME groups was 0.0504 ± 0.005 mg/LV and 0.090 ± 0.004 mg/LV, respectively; melatonin had no effect on hydroxyproline content in soluble collagen fraction (Figure 2B). After four weeks of treatment, the hydroxyproline content in the insoluble collagenous proteins in the control and l-NAME groups was 0.215 ± 0.012 mg/LV and 0.305 ± 0.012 mg/LV, respectively; this was significantly reduced by melatonin (25%, *p* < 0.05) (Figure 2B). The total hydroxyproline content was 0.265 ± 0.017 mg/LV and 0.395 ± 0.015 mg/LV in the control and l-NAME groups, respectively, and it was reduced by melatonin (19%, *p* < 0.05) (Figure 2B).

### 2.3. The Serum Concentrations of Angiotensins and Aldosterone

**Figure 3:**
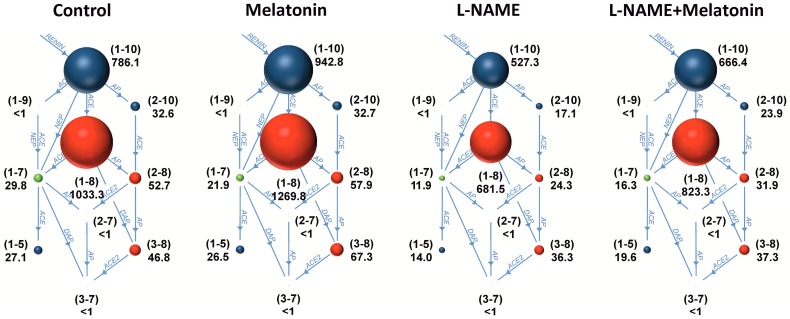
The influence of melatonin (l-NAME + Melatonin) on the serum level of angiotensins represented as pooling of 10 serum samples per each group. *n* = 10 per group.

**Figure 4:**
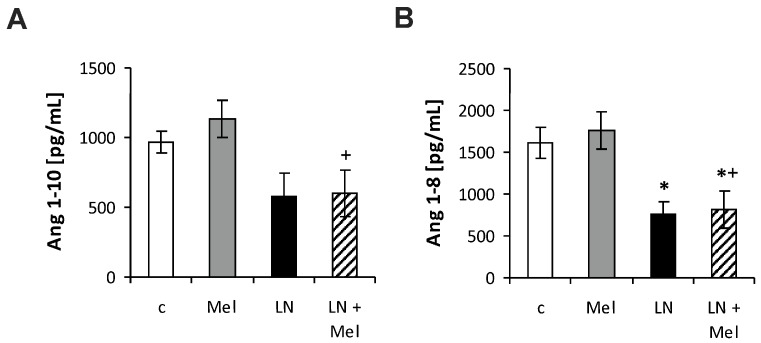
The influence of melatonin (LN + Mel) on the serum level of angiotensin 1–10 (Ang I) (**A**); angiotensin 1–8 (Ang II) (**B**). Wistar controls (c); l-NAME (LN); melatonin (Mel); *n* = 6 per group; * *p* < 0.05 vs. c; + *p* < 0.05 vs. Mel.

**Figure 5:**
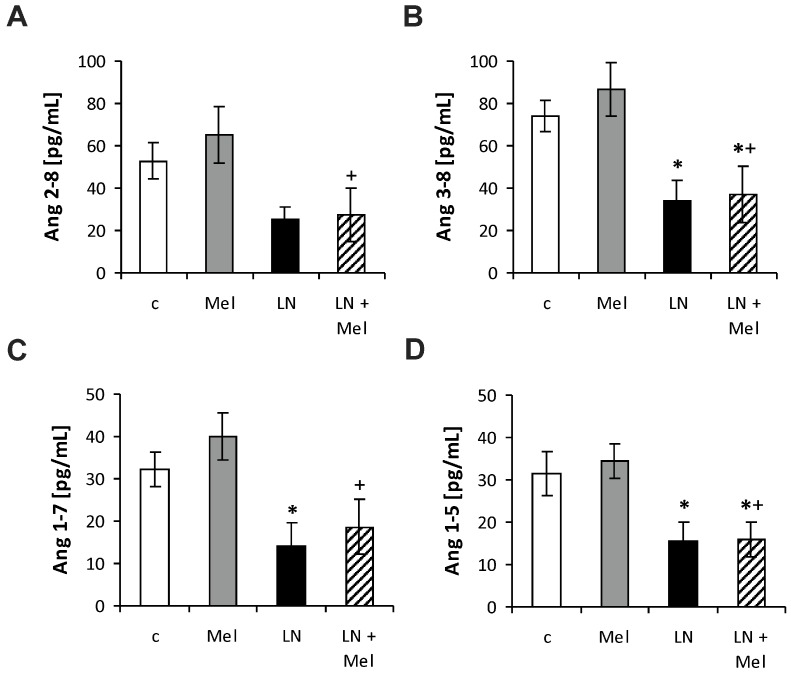
The influence of melatonin (LN + Mel) on the serum level of angiotensin 2–8 (Ang 2–8) (**A**); angiotensin 3–8 (Ang 3–8) (**B**); angiotensin 1–7 (Ang 1–7) (**C**); angiotensin 1–5 (Ang 1–5) (**D**). Wistar controls (c); l-NAME (LN); melatonin (Mel); *n* = 6 per group; * *p* < 0.05 vs. c; + *p* < 0.05 vs. Mel.

**Figure 6:**
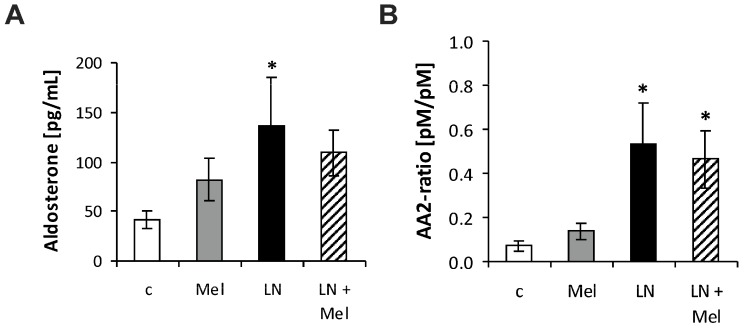
The influence of melatonin (LN + Mel) on the serum level of aldosterone (**A**) and aldosterone/Ang II (AA2) ratio (**B**). Wistar controls (c); l-NAME (LN); melatonin (Mel); *n* = 6 per group; * *p* < 0.05 vs. c.

The serum angiotensins (Ang) concentrations measured by pooling of aliquot samples from all animals per group after four weeks of treatment are shown in Figure 3. After four weeks of treatment, the serum equilibrium level of Ang 1–8 was 1615.44 ± 181.8 pg/mL in the control group and was reduced by l-NAME to 751.9 ± 159.1 pg/mL (52%, *p* < 0.05) (Figure 4B); the level of Ang 1–10 was 967.73 ± 76.63 pg/mL in the control group and was slightly reduced by l-NAME to 579.40 ± 169.0 pg/mL (40%, NS) (Figure 4A); the level of Ang 2–8 was 52.70 ± 8.52 pg/mL in the control group and was slightly reduced by l-NAME to 25.06 ± 5.91 pg/mL (52%, NS) (Figure 5A); the level of Ang 3–8 was 74.11 ± 7.22 pg/mL in the control group and was reduced by l-NAME to 34.30 ± 9.10 pg/mL (54%, *p* < 0.05) (Figure 5B); the level of Ang 1–7 was 32.3 ± 4.08 pg/mL in the control group and was reduced by l-NAME to 13.98 ± 5.82 pg/mL (57%, *p* < 0.05) (Figure 5C); the level of Ang 1–5 was 31.64 ± 5.20 pg/mL in the control group and was reduced by l-NAME to 15.39 ± 4.66 pg/mL (52%, *p* < 0.05) (Figure 5D). Neither of the angiotensin’s levels in the l-NAME group was influenced by melatonin. The serum concentration of aldosterone was 41.50 ± 9.00 pg/mL in the control group and was increased by l-NAME to 136.81 ± 48.59 pg/mL (230%, *p* < 0.05); melatonin only slightly reduced the aldosterone concentration in the l-NAME group (20%, NS) (Figure 6A). The aldosterone/Ang II (AA2) ratio was 0.07 ± 0.02 in the control group and was increased by l-NAME to 0.53 ± 0.18 (657%, *p* < 0.05); melatonin did not influence AA2-ratio (Figure 6B).

## 3. Discussion

Chronic administration of l-NAME is associated with increased SBP and LV hypertrophy development and enhancement of hydroxyproline in soluble, insoluble and total collagenous fractions in the LV. l-NAME treatment resulted in the reduction of serum Ang 1–8 (Ang II), Ang 3–8, Ang 1–7 and Ang 1–5 levels and also an enhancement of aldosterone level and aldosterone/angiotensin II ratio. Melatonin reduced SBP and the concentration and content of LV hydroxyproline in insoluble and total collagen fractions in l-NAME group. These hemodynamic and structural changes induced by melatonin were associated with the maintained reduction of the Ang II level.

**Figure 7:**
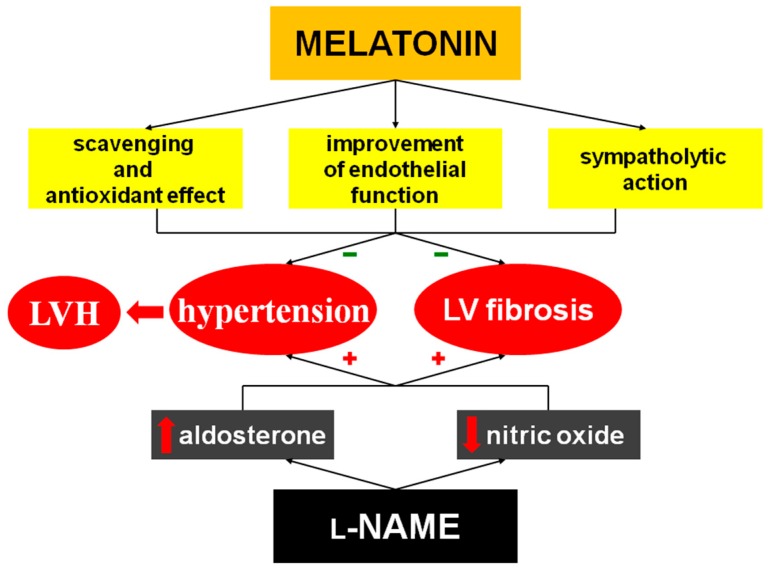
Potential mechanisms of protective effect of melatonin in l-NAME-induced hypertension. Chronic l-NAME administration induces hypertension development, fibrosis of the left ventricle (LV) and via increased hemodynamic load left ventricular hypertrophy (LVH) develops. Melatonin reduces free radical burden by its antioxidant and scavenging effect, improves endothelial function and exerts central sympatholytic effect. These actions of melatonin reduce hypertension development and fibrosis in the LV. Since the effect of melatonin on blood pressure is only mild, LVH remains unaffected.

The important issue is the dose of melatonin that should be used to counteract the pathological alterations induced by l-NAME. The dose 40 mg/kg/day of l-NAME in this experiment was chosen based on our previous studies [3,4,22,23,24,25] and it yielded expected hemodynamic and structural alterations. For melatonin, the dose 10 mg/kg/day used in our previous experiments was shown to lower increased blood pressure and protect the cardiovascular system against the deleterious effects of increased hemodynamic load. On the other hand, in our previous pilot studies, a higher dose of melatonin (30 mg/kg/day) demonstrated obvious sedative effect. Thus, the dose 10 mg/kg/day of melatonin is effective, tolerated and the effects observed in this study can be well compared with the data of previous experiments.

l-NAME-induced hypertension has become one of the most attractive models of hemodynamic overload. In this “NO-deficient” hypertension, NO-synthase activity is inhibited and the cGMP level is suppressed in several investigated organs. The lower nitric oxide production is associated with hypertension development and the structural remodeling of the LV, aorta, kidney and brain [3,4,5,22,26]. Reduced NO availability leads to the hypercontractility of various parts of the vascular bed, particularly the renal artery [6]; this results in an increased renin release. The activation of the renin-angiotensin system along with the depressed NO production is considered the principal neurohumoral disorder participating in hemodynamic and structural alterations in l-NAME-treated rats; treatment with angiotensin converting enzyme (ACE)-inhibitors prevents or reverses these changes [3,4]. Ang II induces the remodeling of variable cardiovascular organs through the vasoconstrictive effect increasing both preload and afterload and by its direct pro-proliferative action [27,28,29,30] (Figure 7).

The situation with the RAAS, however, is considerably more complex than previously suspected [31]. In addition to Ang II, several other peptides (which are downstream metabolites of Ang II) should be considered [32]. ACE2 converts Ang II to Ang 1–7, which seems to have a protective feature in the cardiovascular system. Ang 1–7 has a counteracting effect against Ang II-induced vasoconstriction, inflammation, and cellular growth signaling at the level of the heart and blood vessels under conditions of hypertension, myocardial remodeling and heart failure or stroke [33,34,35,36]. It also seems that Ang 1–5, the scission product of Ang 1–7, could provide cardiovascular protection through the stimulation of the atrial natriuretic peptide release via MAS receptors [37]. These substances may be a therapeutic target or direct cardiovascular protectives. The administration of Ang 1–7 to mice with type 2 diabetes reduced cardiomyocyte hypertrophy, inflammatory cell infiltration and fibrosis and increased blood vessel number in the heart tissue [34] and prevented the development of hypertension and end-organ damage in l-NAME-treated spontaneously hypertensive rats [38]. Thus, these peptides might be of therapeutic value for individuals with reactive heart hypertrophy and fibrotic rebuilding. Herein, l-NAME induced hypertension and fibrosis of the left ventricle. Although Ang II is generally considered to be a principle player in the development of undesirable alterations in the cardiovascular system during the hemodynamic overload [3,30], in our experiment the serum equilibrium Ang II level was reduced in the l-NAME group. Thus, the activation of systemic Ang II production seems not to be consistent with the development of pathologic alterations in a state with NO-deficiency. Moreover, Ang 1–7 and Ang 1–5, which are potentially protective, were also decreased in the systemic circulation and their deficit may have participated in the development of alterations during l-NAME administration. However, these downstream metabolites are produced in much lower amounts than Ang II.

The serum level of aldosterone was, in contrast, significantly enhanced in the l-NAME group in the current study. Based on previous findings, aldosterone produced in the adrenal cortex via the stimulation of AT1 receptors plays one of the principle roles in the fibrotic remodeling of the LV during hemodynamic overload [39,40] or in heart failure [41,42]. Although the original data regarding an aldosterone dominant action in the development of cardiovascular alterations were achieved in aldosterone-infused rats fed with high salt diet [39,40], data has emerged that mineralocorticoid receptor activation is a key player in the development and maintenance of cardiac and vascular remodeling in a broad spectrum of cardiovascular pathologies [43,44]. Indeed, the addition of the aldosterone receptor inhibitor to the standard treatment remarkably reduced mortality in the population with heart failure in the RALES [41] and EPHESUS [42] trials. We suggest that l-NAME-induced NO-deficient hypertension is another condition, where aldosterone could be the principle pathophysiological alteration resulting in the development of hypertension, LV hypertrophy and collagen remodeling.

Our findings in l-NAME hypertension are partially in concert with previously published findings. While increased plasma renin activity was reported by several laboratories [7,45], and local ACE activity was stimulated in the LV and aorta, serum ACE activity was not increased in l-NAME-induced hypertension [7]. On the other hand, an increase in aldosterone concentration was observed in this model and myocardial fibrosis induced via NOS blockade was supposedly induced by the elevated aldosterone level via the increased AT1 receptor number in the adrenal gland [46]; this is supported by our observed increase in the AA2-ratio. Moreover, administration of spironolactone improved NO production and reduced hypertension and left ventricular remodeling in l-NAME-hypertensive rats [47]. Aldosterone production is mainly regulated by Ang II and it is reasonable to expect its decrease along with the reduction of Ang II levels. However, there are several pathways which could explain the fact that aldosterone may be produced relatively independently of Ang II synthesis. First, besides Ang II, aldosterone secretion is modified by a variety of other factors such as adrenocorticotropin, atrial natriuretic peptide or K^+^ and Mg^2+^ levels [48]. Recent findings even indicate that leptin, the adipocyte-derived hormone, may increase the aldosterone production in obese individuals contributing to the development of hypertension [49]. Second, the number of AT1 receptors in the zona glomerulosa of the suprarenal cortex may be of importance. Usui et al. [46] suggested that observed increased aldosterone production in the l-NAME-model may be determined by increased AT1 receptor expression in the suprarenal cortex. Supposedly, increased number of AT1 receptor in the suprarenal gland may have counterbalanced the decreased level of Ang II regarding the aldosterone production in our experiment. Third, a number of papers indicate that NO inhibits aldosterone secretion in glomerulosa cells through a cGMP-independent mechanism [50,51], potentially via direct reduction of steroidogenesis in zona glomerulosa or by downregulation of AT1 receptors [52]. Indeed, l-NAME administration increased the level of aldosterone even 50-fold [53] independently on renin or Ang II level [54]. Thus, not Ang II but rather the enhancement of the systemic aldosterone level should be considered to play a pivotal role in the end-organ damage in NO-deficient rats considering previous findings [55] and also the current results.

Besides angiotensin and aldosterone, sympathetic nervous system and baroreflex-induced alterations of blood pressure (BP) should be considered. Acute inhibition of the NO-synthesis by i.v. injection of l-NAME induced significant rise in BP and baroreflex sensitivity. Although in sympathectomised rats the BP response after l-NAME was observed, baroreflex sensitivity augmentation did not occur. The authors suggested that the reversal of cardiac autonomic control attenuation in sympathectomised l-NAME-treated rats reflected an important role of sympathetic innervations in the acute l-NAME-induced hypertension [56]. However, according to our results it may be hypothesized that under chronic l-NAME-treatment the situation may be different. Increased SBP, achieved potentially via reduced NO production by l-NAME and increased aldosterone secretion, might inhibit the sympathetic activity via the preserved baroreflex mechanism, thus reducing the tone of the renal artery and renin release with attenuation of angiotensin production.

Melatonin is produced in the pineal gland and a variety of other organs [57]. One function of melatonin is to modulate circadian rhythmicity of a number of physiological functions [8,58], melatonin exerts complex antioxidant effects both intra- and extracellularly [18,59,60] and improves endothelial vasodilatory function via the enhancement of NO bioavailability both in peripheral tissues and in the brain [11,61]. As a result, melatonin and its metabolites protect the cardiovascular system in terms of antihypertensive and antiremodeling effects [62,63,64,65,66,67]. Indeed, melatonin reduced fibrosis in spontaneously hypertensive rats [68], l-NAME-induced hypertension [69], continuous-light- [70] and continuous light + l-NAME-hypertension [71] and in isoproterenol-induced heart failure [72]. It was recently proposed that melatonin is likely an important antifibrotic agent in all organs [66]. It was repeatedly revealed that the antifibrotic effects of several substances were related to the modification of the neurohumoral balance, yet the data on melatonin interference with the RAAS are missing. In the current study, melatonin did not have a significant effect on angiotensin II, aldosterone or on potentially protective substances Ang 1–7, Ang 1–5 or Ang 3–8 and it does not seem that the cardio-protective effect of melatonin was achieved by the interference with the RAAS. However, melatonin may reduce blood pressure via several potential ways. This indolamine was shown to reduce free radical burden in variable models of experimental hypertension in our laboratory [68,72,73], including l-NAME- and continuous light + l-NAME-induced hypertension [25,26,69]. In our previous experiments, the reduction of oxidative stress by melatonin was associated with enhanced NOS activity [26,73], attenuation of endothelium-derived constricting factor concentration and reduction of vascular wall tension [26]. Thus, although we did not measure the parameters characterizing NO-production in the current study, our previous experiments indicate that blood pressure reduction by melatonin and its antiremodeling effect may be associated with the improvement of endothelial function in l-NAME-hypertension (Figure 7).

Besides its effect on vascular system, melatonin may exert part of its antihypertensive action via interaction with the central nervous system. The physiology of melatonin is tightly bound with the sympathetic system. On the one hand, the melatonin release is controlled by sympathetic afferentation to the pineal gland, involving the mutual interaction of light/darkness with the retina, suprachiasmatic nucleus (SCN), paraventricular nucleus and stimulation of pineal β1- and α1-adrenergic receptors [74,75]. On the other hand, the GABA-ergic signaling in neurons from SCN to variable parts of the brain and ventrolateral medulla may be modulated by melatonin activity providing a protective mechanism against excessive sympathetic excitation [76]. Indeed, chronic administration of melatonin reduced BP, heart rate, improved β-adrenergic receptor function and baroreflex in spontaneously hypertensive rats [77]. In healthy young men, melatonin reduced SBP, pulse wave velocity along with the reduction of noradrenalin levels [78]. It seems reasonable to suppose that the inhibition of the sympathetic tone on the central or vascular level might participate in the antihypertensive and organ-protective action of melatonin also in the l-NAME-model of hypertension.

The preserved low level of Ang II despite the SBP reduction by melatonin is an interesting finding. We hypothesize that the effect of melatonin on arterial blood pressure need not necessarily result in the reduced renal perfusion, potentially by virtue of improved NO-availability in the renal artery which might counterbalance the systemic blood pressure reduction and maintain kidney perfusion. Moreover, renin release is controlled by a number of neurohumoral stimuli including sympathetic nervous system or oxidative load, both being suppressed by melatonin [11,28].

We conclude that l-NAME-induced hypertension is associated with a reduced level of Ang II and its downstream metabolites and with an increased serum concentration of aldosterone and AA2-ratio. Melatonin does not change the level of angiotensins and aldosterone in NO-deficient hypertension. It is suggested that melatonin exerts its protective effects without affecting the RAAS.

## 4. Materials and Methods

### 4.1. Animals and Treatment

Male adult (three-month-old) Wistar rats (Department of Toxicology and Laboratory Animals Breeding, Dobra Voda, Slovakia) were randomly divided into four groups (10 per group): untreated controls, rats treated with melatonin (10 mg/kg/day), rats treated with l-NAME (40 mg/kg/day), and rats treated with l-NAME + melatonin. l-NAME and melatonin were dissolved in drinking water and their concentration was adjusted to daily water consumption to ensure the correct dosage. The natural water consumption was 12–13 mL/100 g of body weight. To ensure that all of water with dissolved melatonin was actually consumed by a particular rat, only 10 mL/100 mg of water-melatonin solution was offered. The solution was prepared as follows: 10 mg of melatonin was dissolved in 100 mL of water, while no additional substance was added to dissolve the substance. Rats were housed in individual cages at 22–24 °C and fed a regular pellet diet *ad libitum*. All experimental procedures were carried out in accordance with the Guide for the Care and Use of Laboratory Animals published by the US National Institutes of Health (NIH Publication No. 8523, revised 1996). The experiment was approved by the ethical committee of the Institute of Pathophysiology, Faculty of Medicine, Comenius University, Bratislava, Slovakia (approval number: 1306/14-221).

Systolic blood pressure was measured each week by noninvasive tail-cuff plethysmography (Hugo-Sachs Elektronic, Freiburg, Germany) during five days of the week (from Monday to Friday), always from 7:00 to 9:00 a.m. After four weeks, rats were euthanized and their body weight (BW), heart weight, and left ventricular and right ventricular weights (LVW and RVW) were determined and their relative weights (LVW/BW and RVW/BW ratio) were calculated. Left ventricular samples were frozen at −80 °C and later used for the determination of hydroxyproline concentrations. Unless otherwise stated, all chemicals were purchased from Sigma Chemical Co. (Deisenhofen, Germany).

### 4.2. Determination of Hydroxyproline

The samples from the left ventricle were treated stepwise with different buffers as described previously [79]. The soluble collagenous proteins were extracted with 0.5 mol/L CH_3_COOH-pepsin buffer (at 4 °C) and the remaining insoluble collagenous proteins with 1.25 mol/L NaOH (20 min at 105 °C). Hydroxyproline concentration (a marker of fibrosis) was estimated in both collagenous fractions using spectrophotometry at 550 nm [80]. The hydroxyproline content was expressed in mg per total weight of the LV. Ten cardiac tissue samples were used for analysis of hydroxyproline.

### 4.3. Angiotensins and Aldosterone Analyses

The qualitative equivalence of circulating and equilibrium angiotensin levels in rats has been documented and established with the equilibrium levels providing higher sensitivity compared to “snap shot” measurements in plasma treated with various protease inhibitors. Therefore equilibrium angiotensin peptide concentrations and aldosterone levels were determined by mass spectrometry in the serum samples as described previously [32,81,82]. Briefly, the conditioned serum was equilibrated at 37 °C for 30 min followed by the stabilization of equilibrium peptide levels. The stabilized samples were further spiked with stable isotope-labeled internal standards for each angiotensin metabolites (Ang I, Ang II, Ang 1–7, Ang 1–5, Ang 2–8 and Ang 3–8) and aldosterone at concentrations of 200 pg/mL and 500 pg/mL, respectively. Following C18-based solid-phase extraction, the samples were subjected to LC-MS/MS analysis using a reversed-phase analytical column (Acquity UPLC^®^ C18, Waters Corp., Milford, MA, USA) operating in line with a XEVO TQ-S triple quadrupole mass spectrometer (Waters Corp.) in MRM mode. Internal standards were used to correct for peptide recovery of the sample preparation procedure for each angiotensin metabolite in each individual sample. Ang peptide concentrations were calculated considering the corresponding response factors determined in appropriate calibration curves in the original sample matrix, on the condition that integrated signals exceeded a signal-to-noise ratio of 10. Sample of 6 animals were used for analysis of angiotensin and aldosterone.

### 4.4. Statistical Analyses

Results are expressed as mean ± S.E.M. A one-way, two-tailed analysis of variance (ANOVA) and the Bonferroni test were used for statistical analysis of SBP, BW, ventricular weights and hydroxyproline concentration and content in the LV and LSD (Fisher’s least significant difference) test was used for statistical analysis of RAAS data. The differences were considered significant if the *p*-value was <0.05.

## 5. Limitations

The most reliable information regarding the efficiency of absorption of the administered substances is to measure their plasmatic concentrations. The investigation of l-NAME or melatonin plasmatic concentrations was beyond our technical possibilities. However, based on this and several previous experiments with l-NAME and/or melatonin [3,22,25,26,71,83], it may be supposed that both substances were absorbed reliably, since hemodynamic and target organ effects of both substances were clearly demonstrated: gradual blood pressure increase along with fibrotic remodeling of the LV in l-NAME group and attenuation of SBP increase and fibrotic remodeling of the LV when melatonin was applied simultaneously with l-NAME.

The measurement of angiotensins and aldosterone in samples from all ten investigated animals is desirable. Indeed, we have measured and reported angiotensins/aldosterone levels in the pools of all investigated animals in each particular treatment group (obtaining thus a physical average of the levels in each group). To assess the intragroup variability (needed for statistical analysis), we have performed measurements in 6 randomly selected (for cost effectiveness) individual animals from each group. Nevertheless, the average values obtained from 6 and 10 animals were comparable and statistically significant differences were obtained from the analyses of 6 animals already documenting sufficient statistical power.

## Figures and Tables

**Table 1:** The body weight (BW), right ventricular weight (RVW), relative RVW (RVW/BW) and left ventricular weight (LVW) in control rats (c), rats treated with melatonin (Mel), with l-NAME (LN), with l-NAME and melatonin (LN + Mel) (*n* = 10 per group).

	BW (g)	LVW (mg)	RVW (mg)	RVW/BW (mg/g)
c	346.50 ± 6.19	425.30 ± 14.05	164.50 ± 6.08	0.48 ± 0.03
Mel	318.00 ± 7.50	427.00 ± 17.08	160.00 ± 8.40	0.50 ± 0.03
LN	318.50 ± 7.64	461.90 ± 17.98	155.00 ± 7.04	0.49 ± 0.03
LN + Mel	304.00 ± 11.57 *	457.50 ± 22.53	158.60 ± 9.50	0.52 ± 0.03

Values are mean ± S.E.M., * *p* < 0.05 vs. c.
